# Carbon chain elongation microorganism stimulates caproate production from ethanol and acetate under applied voltage regulation

**DOI:** 10.3389/fmicb.2025.1597990

**Published:** 2025-06-18

**Authors:** Jing Li, Xing Luo, He Liu, Xuedong Zhang, Hao Tan, Xiaolong Xiong

**Affiliations:** ^1^Sichuan Institute of Edible Fungi, Sichuan Academy of Agricultural Sciences, Chengdu, China; ^2^Institute of Agricultural Resources and Environment, Sichuan Academy of Agricultural Sciences, Chengdu, China; ^3^School of Environment and Ecology, Jiangnan University, Wuxi, China; ^4^Jiangsu Collaborative Innovation Center of Technology and Material of Water Treatment, Suzhou University of Science and Technology, Suzhou, China

**Keywords:** carbon chain elongation, optimal system, caproate, metabolism pathways, microbial community

## Abstract

Carbon chain elongation has been an innovative process for the synthesis of medium-chain fatty acids (MCFAs). Among them, caproate is a vital multi-functional one. To enhance the synthesis efficiency of caproate, the growth conditions of carbon chain elongation microorganisms need to optimize to develop an ideal niche, ultimately enhancing the production of caproate. In this study, the microbial enrichment of carbon chain elongation was obtained and the optimal system of carbon chain elongation was constructed. The optimal condition for carbon chain elongation was achieved at a pH of 7.00, an ethanol/acetic acid carbon molar ratio of 4:1, and a voltage of 0.7 V. The result showed that the concentration of caproate in the optimal group increased by 83.09% in comparison to the control group. Subsequently, compared with the initial microbial community structure, the relative abundance of microorganisms changed greatly in the optimal system, including *Clostridium_sensu_stricto_12*, *Christensenellaceae_R-7_group*, *Anaerofilum*, *Clostridium_sensu_stricto_7*, and *Intestinimonas*. Additionally, functional prediction analysis revealed that the optimal system enhanced amino acid metabolism (alanine, aspartate, and glutamate), carbon metabolism (CoA biosynthesis), and energy metabolism by 33.66, 30.42, and 17.05%, respectively. Besides, both the fatty acid biosynthesis (FAB) and reverse *β* oxidation (RBO) pathways were enhanced in optimal system. This study elucidates a novel mechanistic insight into the efficient microbial synthesis of caproate through carbon chain elongation pathways, demonstrating how applied voltage regulation can significantly enhance the bioproduction of MCFAs from simple substrates such as ethanol and acetate. Furthermore, this work presents a sustainable and energy-efficient strategy for caproate production, reducing reliance on fossil-derived precursors.

## 1 Introduction

To date, the development of the global economy has heavily dependent on petroleum refining, which causes more and more environmental, materials, and energy issues. Recently, carbon chain elongation and novel biofuel cell have been regarded as a novel and effective strategy to alleviate above issues (Bhatia et al., 2018; Aghaie et al., 2018; Kondaveeti et al., 2022; Gupta et al., 2024). As a secondary fermentation process, carbon chain elongation can be used for the production of medium chain fatty acids (C6-C12, MCFAs) utilizing the ethanol and short chain fatty acids (SCFAs) as the substrates (Wu et al., 2019; Jiang et al., 2022; Van Eerten-Jansen et al., 2013; Jiang et al., 2019; Agler et al., 2011; Ameen et al., 2019). MCFAs have broad applications across various industries. In agriculture, they are utilized as “green” antibiotics, while in the food industry, they serve as food additives. Additionally, MCFAs are key raw materials for chemical products, such as precursors to plasticizers, lubricants, and surfactants. In recent years, MCFAs have demonstrated significant potential as fuel precursors in the renewable energy sector, including applications in rubber, diesel, and aviation fuels. As one typical MCFAs, caproate can be utilized for multifarious applications, including antimicrobial agents, flavor additives, and feedstock (Reddy et al., 2018). Meanwhile, the production of caproate *via* microbial carbon chain elongation is a process that depends on electron donors. Ethanol and electrodes are commonly used as electron donors. Different molar ethanol/acetate ratio and voltage affect the carbon chain elongation reaction. Some studies indicated that excessive ethanol/acetate ratio and high voltages could inhibit the activity of microorganisms and formation of microorganisms’ biofilm (Wu et al., 2019; Wang and Yin, 2022; Leng et al., 2017; Wu et al., 2021). Among them, carbon chain elongation microorganisms play a vital role in MCFAs production, such as *Clostridium kluyveri*, *Megasphaera elsdenii*, and *Eubacterium limosum*. Microbial metabolic pathway and electron transfer also affected the occurrence of carbon chain elongation reaction.

Schievano et al. (2016) reported that the application of an imposed voltage influences the microbial metabolism and fermentation environment in either an oxidative or reductive manner. Van Eerten-Jansen et al. (2013) showed that the conversion of acetate and ethanol to MCFAs required voltage and hydrogen evolution reaction assistance. Wu et al. (2021) showed that correlation analysis of cathode parameters at 0.6 V was optimal voltage to fermentation conditions (particularly pH and redox potential) in chain elongation reaction. This study established an efficient method for voltage-regulated bioproduction from acetate. Wu Y. D. et al. (2020), Wu S. L. et al. (2020), and Wu P. et al. (2020) reported that the co-production of MCFAs was improved with the increase of the carbon molar ratio of ethanol to activated sludge, but the maximum concentration of the target product was achieved at the ratio of electron acceptor to electron donor of 1:4. Therefore, in order to increase the production of MCFAs, the optimization of the ethanol/acetate carbon molar ratio and the applied voltage are urgently needed to explore, finally increasing the yield of caproate.

The study has three major aims: (1) to explore the effects of different carbon molar ratios of ethanol/acetic acid and voltage on caproate production in carbon chain elongation; (2) to analyze the redox characteristics, aggregation, and extracellular polymeric substance (EPS) secretion of biofilm microorganisms under optimal system; (3) to reveal the influence of different voltage on chain elongation metabolic pathways and microbial community structure, and function prediction of gene expression. The effects of carbon molar ratios of ethanol/acetic acid and voltage on structural composition of the microbial community, biofilm characterization, and metabolic pathway were elucidated. These findings provide new perspectives on the biotechnological applications of chain elongation for producing value-added products.

## 2 Material and method

### 2.1 Batch experiment

The optimization of parameters of the carbon chain elongation reaction in this study is mainly categorized into two steps. The first step was to explore the influence of the optimal carbon molar ratio of ethanol/acetate on carbon chain elongation microorganisms; the second step was to examine the influence of different voltages on carbon chain elongation microorganisms under the optimal carbon molar ratio of ethanol/acetate. Specific steps are as follows, firstly, the carbon chain elongation enrichment was inoculated into the medium at 10% (V/V) inoculations, followed by the addition of substrates with different molar ratios of ethanol/acetate carbon (6:1, 4:1, 2:1, and 1:1). The initial pH of the reactors was maintained at 7.00, and then dinitrogen gas was employed to purge test bottles for 30 min to retain an anaerobic niche, with subsequent culturation in a shaker at 150 rpm, 36 ± 1°C incubator (Liu et al., 2016). After the optimal ethanol/acetate carbon molar ratio was determined as aforementioned, the second step is as follows: Connect the reactors to the electrochemical workstation, set different voltages (0 V, 0.4 V, 0.7 V, 1 V, and 1.5 V), adjust the initial pH value to 7.00, and then purge the anaerobic bottles for 30 min using dinitrogen gas to maintain an anaerobic environment. During the tests of optimization of the carbon chain elongation process, 2 mL of liquid was taken every day to detect the changes of ethanol and carboxylic acid compounds in the reactors. Each experimental treatment was independently replicated at least three times to ensure reproducibility and validate the reliability of the results.

### 2.2 Biofilm characterization

The morphological structure of the cathodic biofilm was examined using the application of scanning electron microscopy (SEM, Snetar Precision Instruments, Co. Ltd., China). The samples of carbon felt electrodes, each measuring 1 cm × 1 cm, were subjected to pretreatment with ethanol and a 2.5% glutaraldehyde solution prior to analysis *via* SEM (Iga et al., 2024; Chu et al., 2020; Ludovic et al., 2018; Yu et al., 2019). The EPS from cathodic biofilm microorganisms were extracted and diluted with deionized water. The biofilm sample was transferred to a 50 mL centrifuge tube, diluted to 30 mL with 0.6% NaCl solution, and centrifuged at 1,660 g for 10 min at 4°C to remove residual medium. The pellet was resuspended in 0.6% NaCl solution to restore the original volume, followed by centrifugation at 4,000 g for 15 min at 4°C. The supernatant was collected, filtered through a 0.45 μm aqueous polyether sulfone (PES) membrane, and the resulting filtrate was designated as EPS (Han et al., 2023). The corresponding emission wavelengths of 200–550 nm and excitation wavelengths of 200–450 nm were selected.

The redox properties of cathodic biofilm were assessed using a cyclic voltammetry system (CV, Zhong Deke Yi Electric Gas Technology Co. Ltd., China). The CV was conducted under the following parameters: a scan rate of 1 mV/s, with scanning range of the voltage set from +1.2 V to-1.2 V (Kousar and Laurila, 2024).

### 2.3 Microbial community and analysis

16 s rRNA microbial community was employed to detect the microbial community of chain elongation processes. At the end of the operation, according the biofilm distribution on the cathode, three points of biofilm were defined for sampling, and the biofilm microorganism was harvested by shaking and then the three samples were homogenously mixed. The DNA of the samples was extracted utilizing the FastDNA™ Spin Kit (MP bio, U.S). The V3-V4 regions of the 16S rRNA gene were successfully amplified through the application of primers 338F (50-ACTCCTACGGGAGGCAGCAG-30) and 806R (50-GGACTACHVGGGTWTCTAAT-30) *via* PCR (Gene Amp 9,700, ABI, USA) (Mao et al., 2005; Aßhauer et al., 2015).

The concentrations of compounds, including acetate, ethanol, butyrate, and caproate were analyzed using gas chromatography (Shimadzu Technology, Co. Ltd., Japan) (Liu et al., 2020; Li et al., 2022).

## 3 Results and discussion

### 3.1 Carbon chain elongation process

The carbon chain elongation process is an important biosynthesis pathway, which mainly consists of electron donor oxidation, Reverse *β* oxidation (RBO), and fatty acid biosynthesis (FAB) pathways. In this process, electron donor is necessary to initiate carbon chain elongation reaction. In general, electron donor is a type of high-energy reducing substances that can provide required electrons, energy, and necessary intermediate products, such as acetyl coenzyme A. In addition, RBO is a typical carbon chain elongation metabolic pathway, and electron donor is an important substance in the carbon chain elongation process. Among them, ethanol and lactate are considered as the most ideal electron donors in the carbon chain elongation process. Therefore, this study mainly investigated the mechanisms involved in carbon chain elongation based on the RBO process of producing caproic and caprylic acids using ethanol as electron donors.

**Figure 1:**
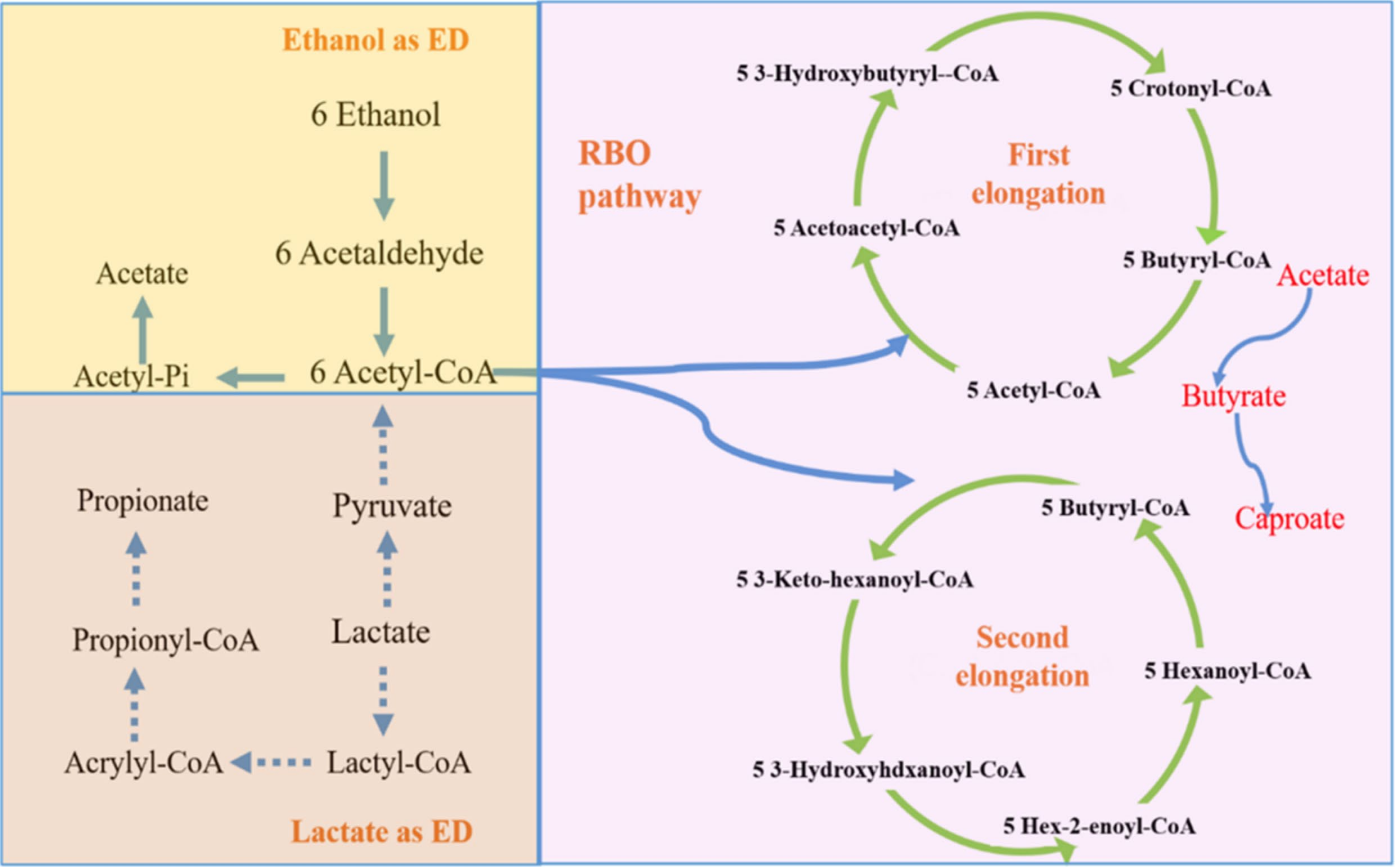
Carbon chain elongation RBO mechanism diagram with ethanol and lactate as electron donors.

Under standard thermodynamic conditions (1 atm, 25°C), it was feasible to produce caproate by carbon chain elongation process regarding ethanol and lactic acid as electron donors (Equations 1, 2 and Figure 1). As ethanol is used as electron donor, ethanol is first oxidized to acetaldehyde by ethanol dehydrogenase, and then acetaldehyde is converted to the important intermediate product (acetyl-CoA). Approximately 1/6 of the acetyl-CoA *via* substrate-level phosphorylation is converted to acetic acid, harvesting energy (Equation 3). Meanwhile, the 5/6 of the acetyl-CoA gets into the RBO pathway. In RBO cycle, acetyl-CoA couples with another molecule of a derivative of coenzyme A, generating a coenzyme A derivative that extends the chain of acids by adding two carbons. First, acetate accepts electron donor to produce butyrate (Equation 4). Later, acetyl-CoA couples with butyryl-CoA to start the second cycle to produce caproate (Equation 5).

When lactic acid serves as an electron donor, it is first oxidized to pyruvic acid by lactate dehydrogenase, and then pyruvic acid is further oxidized to acetyl-CoA, accompanied by the release of CO_2_ and the production of ATP. Similar to ethanol as an electron donor, some acetyl-CoA is also converted to acetic acid (Equation 6). It is worth noting that propionic acid can be generated through the propionate pathway, which competes with the carbon flow for lactic acid chain elongation. However, it is difficult to elongate propionic acid to heptanoic acid. Therefore, in order to achieve a higher recovery rate of MCFAs, the occurrence of the propionate pathway should be limited. As aforementioned, the acetyl-CoA is produced by the oxidation of lactic acid, entering the RBO cycle to generate butyric acid (Equation 7), and then further being elongated to caproic acid (Equation 8).


(1) $$\begin{array}{l} {\text{CH}}_{3}{\text{CH}}_{2}\text{OH}+2{\text{CH}}_{3}{\text{COO}}^{-}+2H_{2}+H^{+}\\ \to {\text{CH}}_{3}{\text{CH}}_{2}{\text{CH}}_{2}{\text{CH}}_{2}{\text{CH}}_{2}{\text{COO}}^{-}+3H_{2}O\; \end{array}$$

(2) $$\begin{array}{l} 2{\text{CH}}_{3}{\text{CH}}_{2}{\text{COOO}}^{-}+{\text{CH}}_{3}{\text{COO}}^{-}+2H^{+}\\ \to {\text{CH}}_{3}{\text{CH}}_{2}{\text{CH}}_{2}{\text{CH}}_{2}{\text{CH}}_{2}{\text{COO}}^{-}+2H_{2}O+2{\text{CO}}_{2}\; \end{array}$$

(3) $${\text{CH}}_{3}{\text{CH}}_{2}\text{OH}+H_{2}O\to {\text{CH}}_{3}{\text{COO}}^{-}+2H_{2}+H^{+}\;$$

(4) $$\begin{array}{l} 6{\text{CH}}_{3}{\text{CH}}_{2}\text{OH}+4{\text{CH}}_{3}{\text{COO}}^{-}\to 5{\text{CH}}_{3}{\text{CH}}_{2}{\text{CH}}_{2}{\text{COO}}^{-}\\ +4H_{2}O+2H_{2}+H^{+}\; \end{array}$$

(5) $$\begin{array}{l} 6{\text{CH}}_{3}{\text{CH}}_{2}\text{OH}+5{\text{CH}}_{3}{\text{COO}}^{-}\to \\ 5{\text{CH}}_{3}{\text{CH}}_{2}{\text{CH}}_{2}{\text{CH}}_{2}{\text{CH}}_{2}{\text{COO}}^{-}+{\text{CH}}_{3}{\text{COO}}^{-}\\ +4H_{2}O+2H_{2}+H^{+}\; \end{array}$$

(6) $${\text{CH}}_{3}{\text{CH}}_{2}{\text{COOO}}^{-}+H_{2}O\to {\text{CH}}_{3}{\text{COO}}^{-}+2H_{2}+{\text{CO}}_{2}\;$$

(7) $$\begin{array}{l} {\text{CH}}_{3}{\text{CH}}_{2}{\text{COOO}}^{-}+{\text{CH}}_{3}{\text{COO}}^{-}+H^{+}\\ \to {\text{CH}}_{3}{\text{CH}}_{2}{\text{CH}}_{2}{\text{COO}}^{-}+H_{2}O+{\text{CO}}_{2}\; \end{array}$$

(8) $$\begin{array}{l} {\text{CH}}_{3}{\text{CH}}_{2}{\text{COOO}}^{-}+{\text{CH}}_{3}{\text{CH}}_{2}{\text{CH}}_{2}{\text{COO}}^{-}+H^{+}\\ \to {\text{CH}}_{3}{\text{CH}}_{2}{\text{CH}}_{2}{\text{CH}}_{2}{\text{CH}}_{2}{\text{COO}}^{-}+H_{2}O+{\text{CO}}_{2}\; \end{array}$$

### 3.2 Impacts of different ethanol/acetate molar ratios on caproate

**Figure 2:**
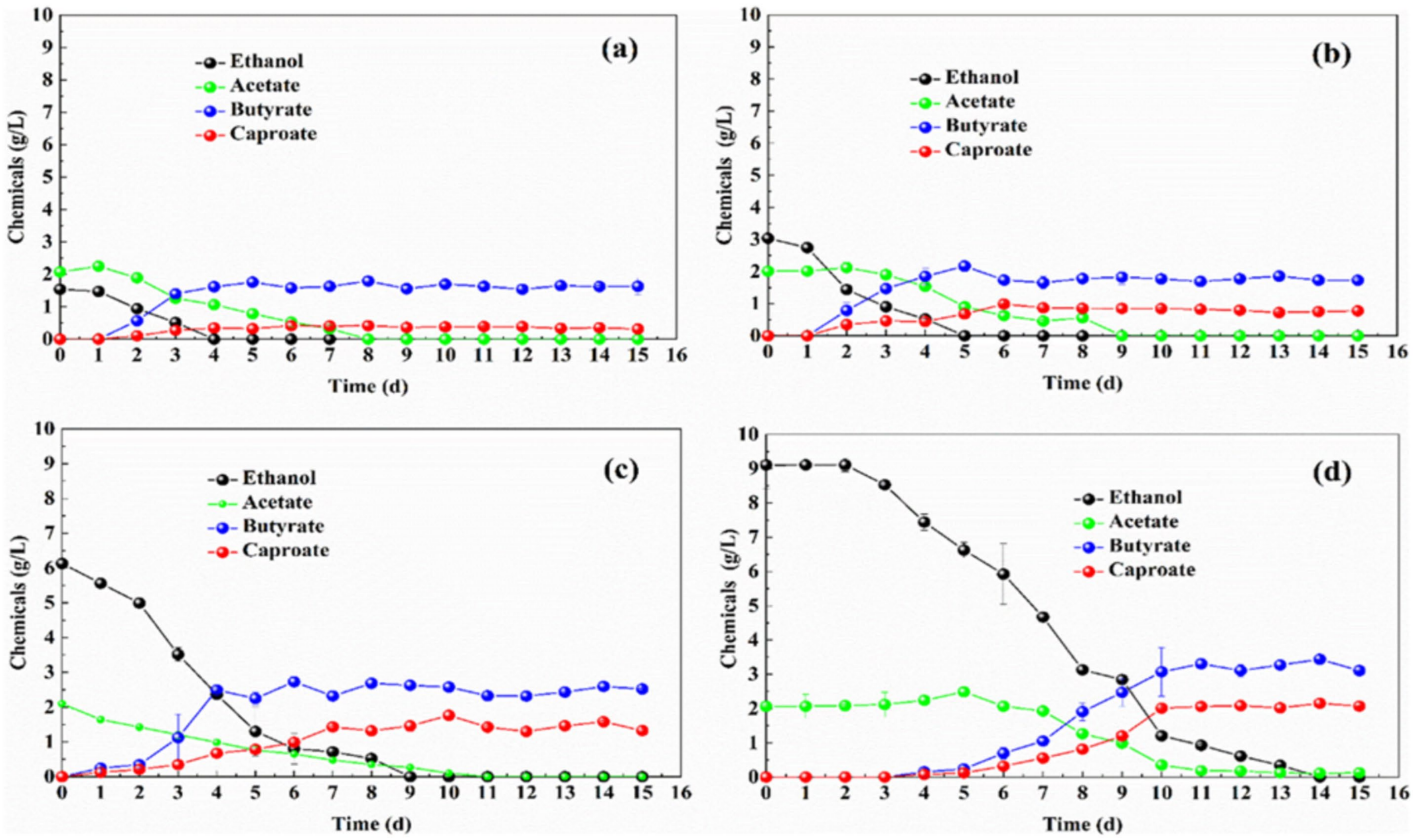
Effect of ethanol/acetate carbon molar ratios on the substrate and products of carbon chain elongation process and the ethanol/acetate carbon molar ratio was 1:1 **(a)**, 2:1 **(b)**, 4:1 **(c)**, and 6:1 **(d)**, respectively.

Firstly, the effects of different carbon molar ratio of ethanol/acetate on the MCFAs production were performed and the changes in ethanol and carboxylic acid concentrations during carbon chain elongation are shown in Figure 2. When the ethanol/acetate carbon molar ratio was 1:1 (Figure 2a) and 2:1 (Figure 2b), ethanol was completely consumed on the 4th and 5th days, respectively, whereas acetic acid was not completely consumed. However, ethanol was completely consumed on the 9th and 14th days when the ethanol/acetate carbon molar ratio were 4:1 (Figure 2c) and 6:1 (Figure 2d). Besides, the ethanol/acetate carbon molar ratio was 6:1 and acetate were almost completely consumed on the 10th day, where ethanol was still left. The results mentioned above showed that the high ethanol/acetate carbon molar ratio made it difficult for the electron donor (ethanol) to be consumed due to the lack of electron acceptor (acetate). As the ethanol/acetate carbon molar ratio was increased from 2:1 to 4:1, the concentration of caproate was raised from 0.78 g/L to 1.33 g/L, and the yield of caproate was increased by 70.51%. When the ethanol/acetate carbon molar ratio was increased to 6:1, although the concentration of caproate was raised to 2.07 g/L, the yield of caproate was only increased by 55.64%. Therefore, caproate yield at the ethanol/acetate carbon molar ratio of 6:1 was reduced by 14.87%, compared to that at the ratio of 4:1.

The results showed that the high carbon molar ratio of ethanol/ acetate could increase the concentration of caproate, but not its yield. Mainly because at the high concentration of ethanol/acetate molar ratio the deficiency of acetate as electron acceptor took place, it would actually lead to the excess of ethanol as electron donor, which was not conducive to the effective use of the substrate. On the 10th day, when the ethanol/acetate carbon molar ratio was 6:1, 1.21 g/L of ethanol and 0.35 g/L of acetate were left. Therefore, in the carbon chain elongation process, manipulating the ethanol/acetate carbon molar ratio at an optimal level was essential to the rapid occurrence of carbon chain elongation. Based on the results, the ethanol/acetate carbon molar ratio of 4:1 was regarded as the optimal ratio in this current study to have high yield of caproate and efficient utilization of either ethanol and acetate.

### 3.3 Effects of different voltages on chain elongation process

**Figure 3:**
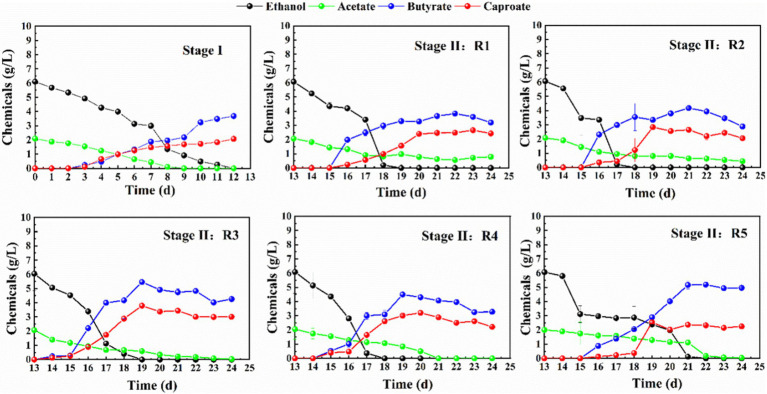
The concentration of ethanol and carboxylic acids during the domestication stage (Stage I, 12 days), alongside the influence of voltage on the production of carboxylic compounds from acetate and ethanol: R1 at 0 V, R2 at 0.4 V, R3 at 0.7 V, R4 at 1 V, and R5 at 1.5 V during 12 days (stage II).

The influences of different voltages on chain elongation process were subsequently performed and the concentration of ethanol and carboxylic acids were detected on a daily basis at Stage I and II. Firstly, acetate and ethanol presented different performances in conversion in the five reactors under different voltage (Figure 3), namely, R1, R2, R3, R4, and R5. In R3 (0.7 V), acetate and ethanol were firstly converted into butyrate 0.23 ± 0.01 g/L, and in R4 (1V) butyrate reached 0.53 ± 0.04 g/L on the 15th day. However, in R1 (0 V), R2 (0.4 V), and R5 (1.5 V) butyrate was detected until the 16th day. On the 19th day, the concentrations of caproate were 2.07 ± 0.11, 2.83 ± 0.03, 3.79 ± 0.14, 3.01 ± 0.06, and 2.54 ± 0.10 g/L in R1, R2, R3, R4 and R5, respectively. The order was as follows: R3 > R4 > R2 > R5 > R1. Consequently, R3 demonstrated the best performance in chain elongation process, which increased by 83.09% in comparison to R1. Caproate was synthesized as a result of the accumulation of butyrate and ethanol at 0.7 V, and the sufficient electron acceptor and electron donor ascertained the carbon chain elongation process (Chu et al., 2020; Xu et al., 2021).

In contrast, under a 1.5 V applied voltage, acetate and ethanol were slowly converted, presumably indicating that the electron transfer capability between solid cathode and microorganisms may have been compromised. Consequently, the analysis of the five reactors indicated that the chain elongation performance was not enhanced as the voltage increased. The research elucidated various mechanisms underlying the influence of voltage on the microbial metabolic activity (Lin et al., 2019; Chen et al., 2018; Wu Y. D. et al., 2020; Wu S. L. et al., 2020; Wu P. et al., 2020). For instance, Lin et al. (2019) demonstrated that an applied voltage of 0.8 V enhanced methane production significantly *via* facilitating interspecies H_2_ transfer. Chen et al. (2018), Wu Y. D. et al. (2020), Wu S. L. et al. (2020) and Wu P. et al. (2020) proposed that the 0.2 V voltage enhanced the cell membranes, permeability and facilitated the electron transfer significantly, thereby improving the redox capacity of microorganism. Hence, the influence of voltage on the fate of the chain elongation process were affected by the microbial metabolic activity, interspecies H_2_ transfer, cell membranes permeability, the reduction power of the cathode (Schievano et al., 2016; Liu et al., 2020; Carvajal-Arroyo et al., 2020), and the ability of extracellular electron transfer (Izadi et al., 2020; Wu Y. D. et al., 2020; Wu S. L. et al., 2020; Wu P. et al., 2020; Hirose et al., 2018).

These mechanisms have been extensively reported in bio-electrochemical systems, wherein the activity of the inoculum and the capacity for electron transfer are influenced by the potential of the cathode (Hirose et al., 2018). Consequently, several conjectures may be proposed regarding the chain elongation system under the modulation of voltage: (1) the conversion of acetate and ethanol represented a synergistic process between the electrode and the inoculum, governed by the catalytic ability of the biocathode; (2) elevated voltages were likely to precipitate the degradation of the cathode reaction niche, leading to the consumption of acetate and ethanol for energy production rather than facilitating chain elongation. To demonstrate these assumptions on regulating acetate and ethanol conversion by manipulating the applied voltage, characterization of biofilm formation was conducted.

### 3.4 Characterization of biocathodes

**Figure 4:**
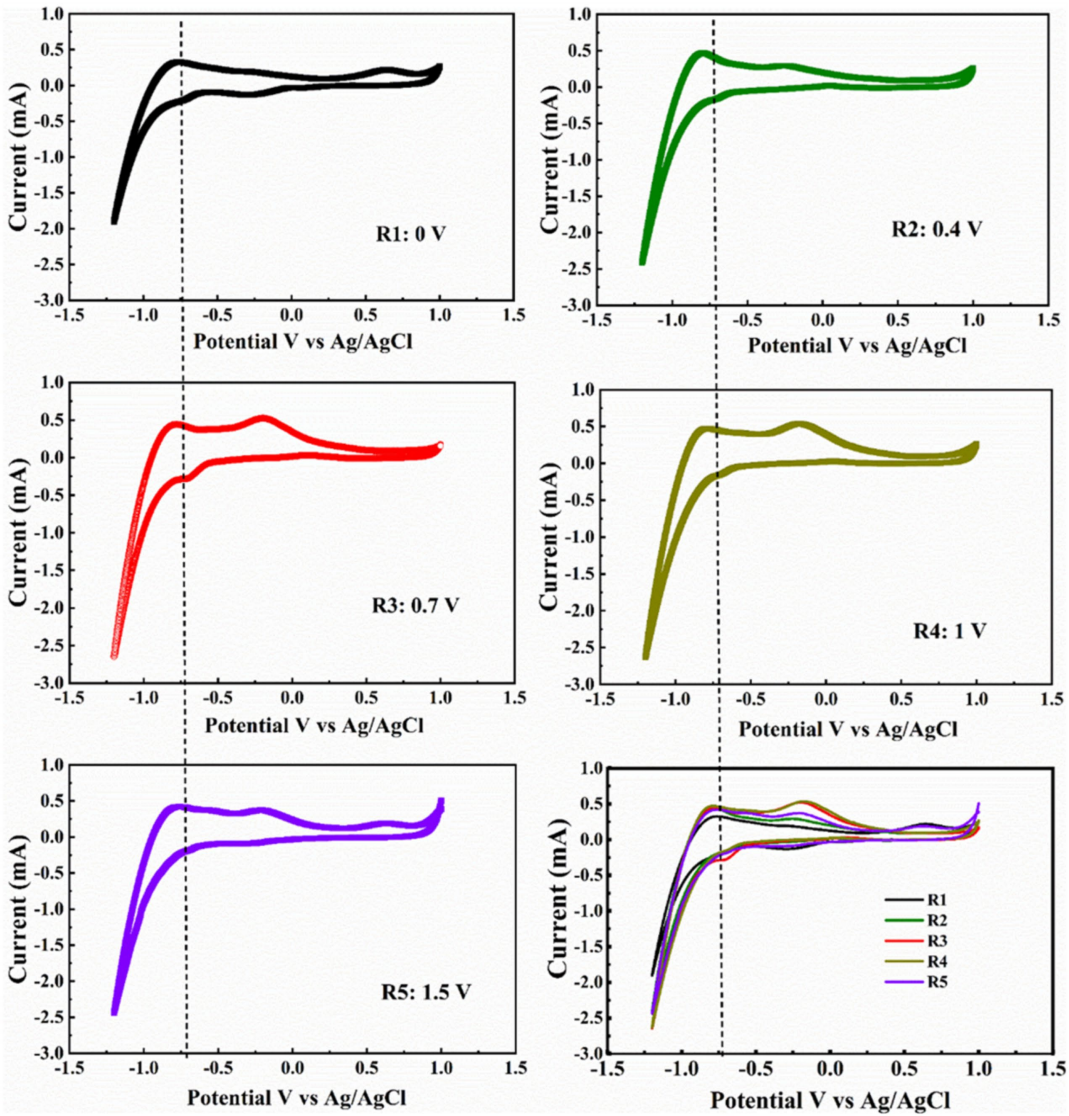
CV of biofilm after stage II operation with different voltage.

The cathode biofilm was collected by the end of the test, and the characterization of the cathode biofilm was measured by CV, SEM, and EPS. Firstly, the impacts of different voltages on the redox characteristics of the cathode biofilm were analyzed by CV (Figure 4). The cathode biofilm of R3 presented an obvious reduction peak (−0.70 V, −0.28 mA). The results of other reactors were as follows: R1 (−0.70 V, −0.13 mA), R2 (−0.70 V, −0.16 mA), R4 (−0.70 V, −0.15 mA), and R5 (−0.70 V, −0.19 mA). Hence, the cathode biofilm of R3 evidently possessed the strongest redox properties. Different voltages affected the carbon chain elongation process mainly through the following three aspects: (1) providing electrons demand for the carbon chain elongation process, (2) regulating the activity of carbon chain elongation microorganisms, and (3) affecting the ability of interspecific H_2_ transfer. It has been reported that low voltage enhances the up-regulation of proteins correlation to electron transfer, thereby improving the redox capacity of microorganisms (Chen et al., 2018; Yu et al., 2017). Therefore, it is suggested that electrons provided by a voltage of 0.7 V might better bond with hydrogen protons to generate more H_2_. In addition, 0.7 V voltage might better regulate the reducing ability of the cathode biofilm microorganisms, which is conducive to the occurrence of carbon chain elongation process.

Secondly, the morphology and aggregation of cathode biofilm microorganisms were observed by SEM. The results showed that less microorganisms were attached to the surfaces of R1, R2 and R5. However, a large number of microorganisms were attached to the surfaces of R3 and R4, and their morphological structure was mainly spherical (Supplementary Figure S1). Among them, R3 presented the highest number of surface-attached microorganisms, while R5 treated biofilm had very few microorganisms attached, probably because the high voltage of 1.5 V was not conducive to the growth of microorganisms.

**Figure 5:**
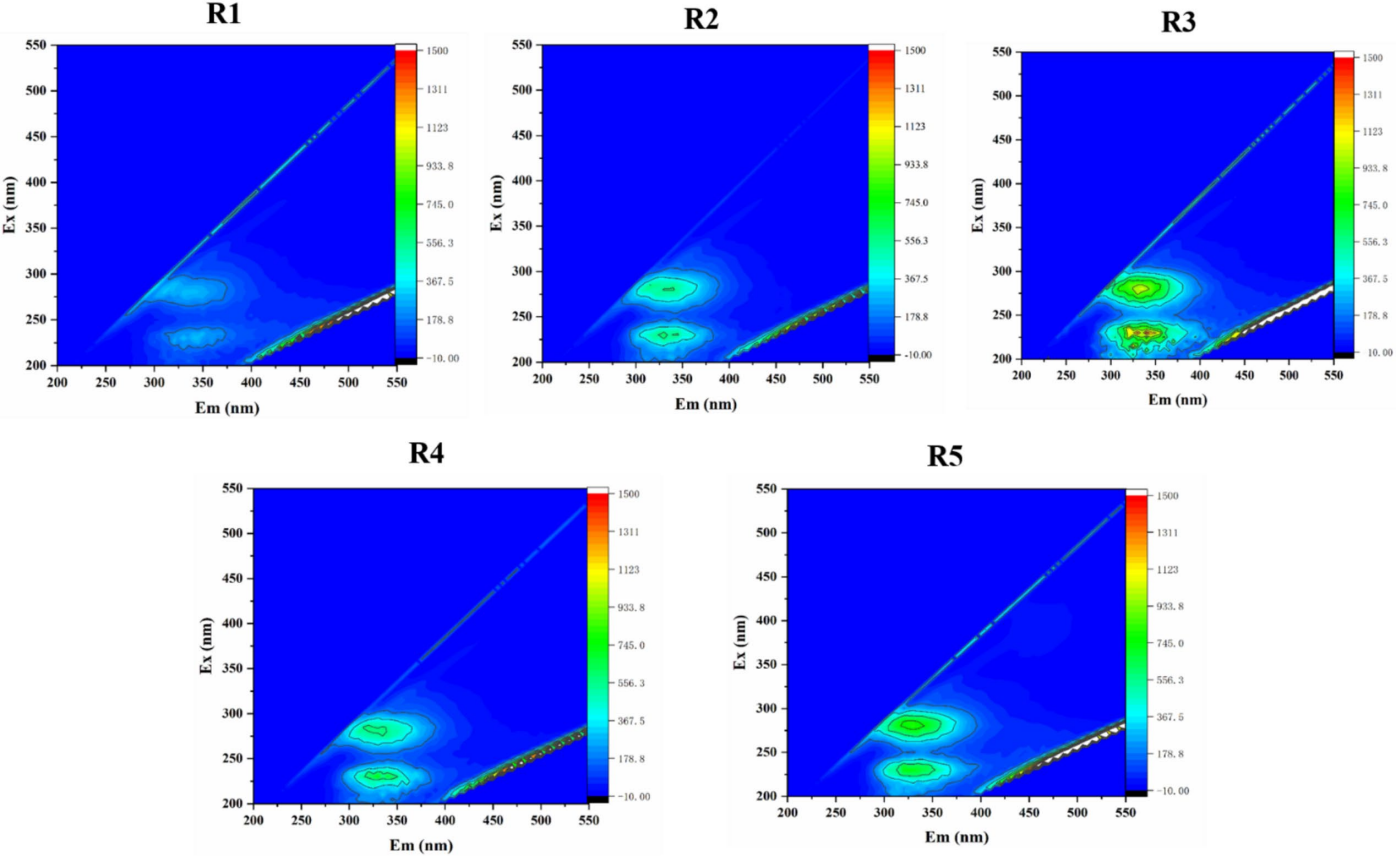
Change of EPS fluorescence spectra at the cathode biofilm of different reactors.

Thirdly, the EPS of cathode biofilm microorganisms was extracted and detected. The fluorescent peaks were divided into five regions. Notably, Regions I, II, and IV were associated with aromatic amino acids and their corresponding proteins. The fluorescence intensity of EPS in Regions I, II, and IV exhibited a marked enhancement under a voltage regulation of 0.7 V (Figure 5 and Supplementary Figure S2). Thus, voltage of 0.7 V demonstrated optimal performance in augmenting the abundance of aromatic amino acids and their associated proteins, ultimately leading to an increase in the fluorescence intensity of EPS.

Based on the results CV, SEM, and EPS aforementioned, the adhesion quantity of microorganisms, redox characteristics, and fluorescent intensity of cathode biofilm did not increase with the elevated voltage, but low voltage was more conducive to the adhesion of cathode biofilm microorganisms, the enhancement of cathode biofilm reduction ability, and fluorescent intensity of cathode biofilm microorganisms. All these benefits seemingly were all attributed cathode biofilm microorganisms, and then the cathode biofilm microorganisms were analyzed under different voltages.

### 3.5 Microbial community analysis

**Figure 6:**
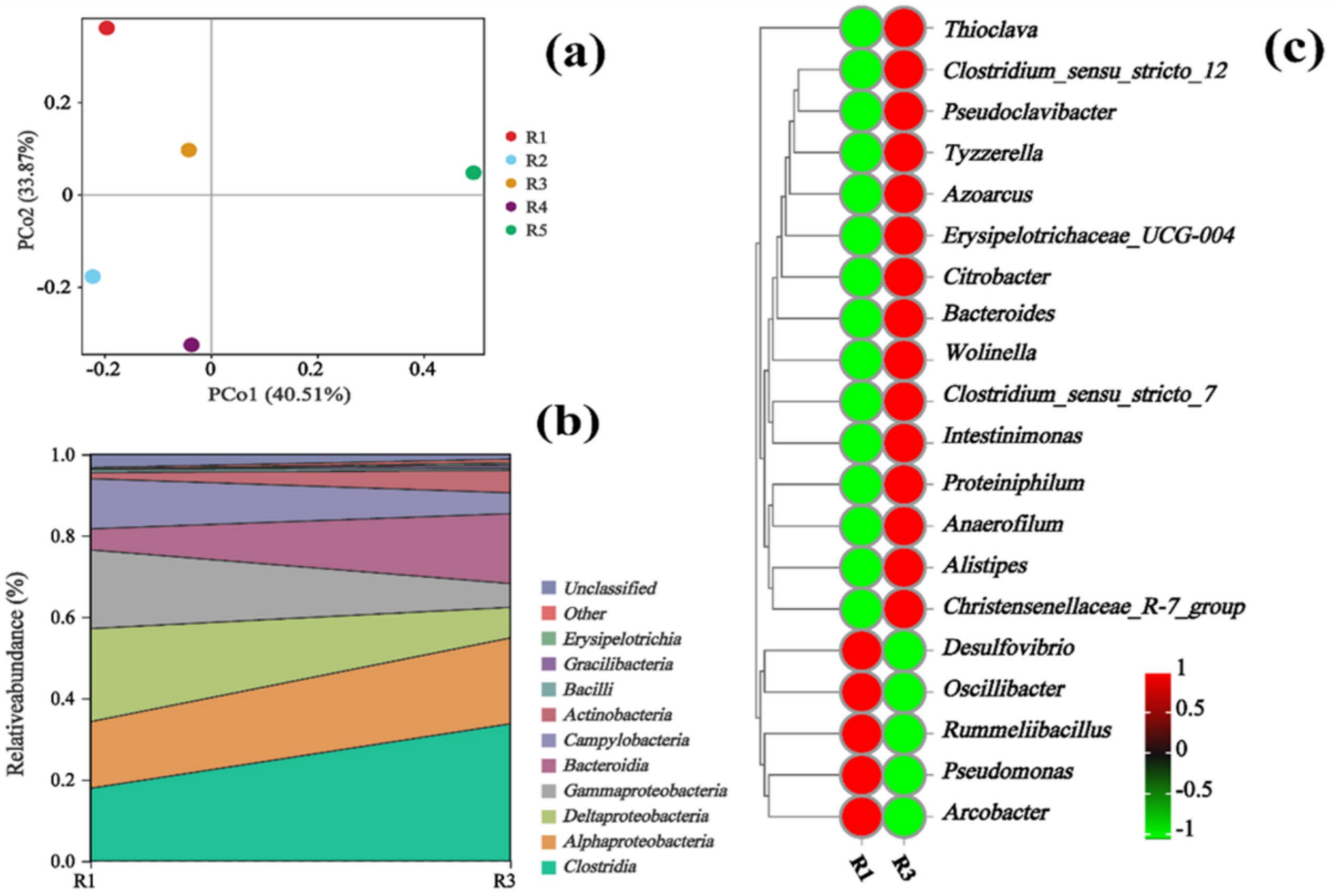
PCoA at the OTU level **(a)**, river distribution map at class level **(b)**, and heatmap at genus level of microbial communities **(c)**.

In order to understand the effects of different voltages on the microbial communities of cathode biofilm, the microorganisms on different cathode biofilm were detected and analyzed. Firstly, the *β* diversity of different reactors was processed by means of Principal coordinates analysis (PCoA), which showed that principal component 1 and principal component 2 accounted for 74.38% of the total microbial population (Figure 6a). The results of PCoA indicated that R1 (0 V), R2 (0.4 V), R3 (0.7 V), R4 (1 V), and R5 (1.5 V) were distributed in different regions, indicating changes in microbial community profile regulated by different voltages. Secondly, at the class level (Figure 6b), the microorganisms on the cathode biofilm were mainly *Clostridia*, *Alphaproteobacteria*, *Deltaproteobacteria*, *Gammaproteobacteria*, and *Bacteroidi*. Among them, *Clostridia* and *Alphaproteobacteria* had a distinction in microbial composition between R1 treatment and R3 treatment. Specifically, the relative abundance of *Clostridia* and *Alphaproteobacteria* from R1 to R3 increased 15.86 and 4.76%, respectively.

Thirdly, the microbial community profile at the genus level of different reactors was shown in Figure 6c. The cathode biofilm microorganisms were mainly composed of *Thioclava*, *Desulfovibrio*, *Oscillibacter*, *Clostridium_sensu_stricto_12*, and *Clostridium_sensu_stricto_7*. Specifically, microorganisms with relatively large changes between R1 treatment and R3 treatment were listed below: *Clostridium_sensu_stricto_12* (0.37–17.84%), *Clostridium_sensu_stricto_7* (0.09–2.49%), *Christensen ellaceae_R-7_group* (0.11–0.50%), *Anaerofilum* (0.18–0.27%), and *Intestinimonas* (0.05–0.34%). Interestingly, all of the microbes aforementioned belong to *Clostridia*. In addition, *Thioclava* (16.22–20.76%) is an important electrochemically active bacterium that plays a variety of important roles in sulfur oxidation, crude oil degradation, and biopower generation (Liu et al., 2017). *Desulfovibrio* (22.90–7.52%) is a typical sulfate-reducing bacterium. It was assumed that BES (C_2_H_4_BrNaO_3_S) was added to the culture medium, and it contains sulfur, which might be used as substrate and energy resource by sulfate-reducing bacteria (Lumppio et al., 2001). Based on the above results, appropriate voltage could not only increase the abundance of chain elongation *Clostridia*, but also enhance some other microorganisms, such as *Thioclava*, finally resulting in the enhancements of the acid production in carbon chain elongation reaction.

### 3.6 Microbial function prediction

**Figure 7:**
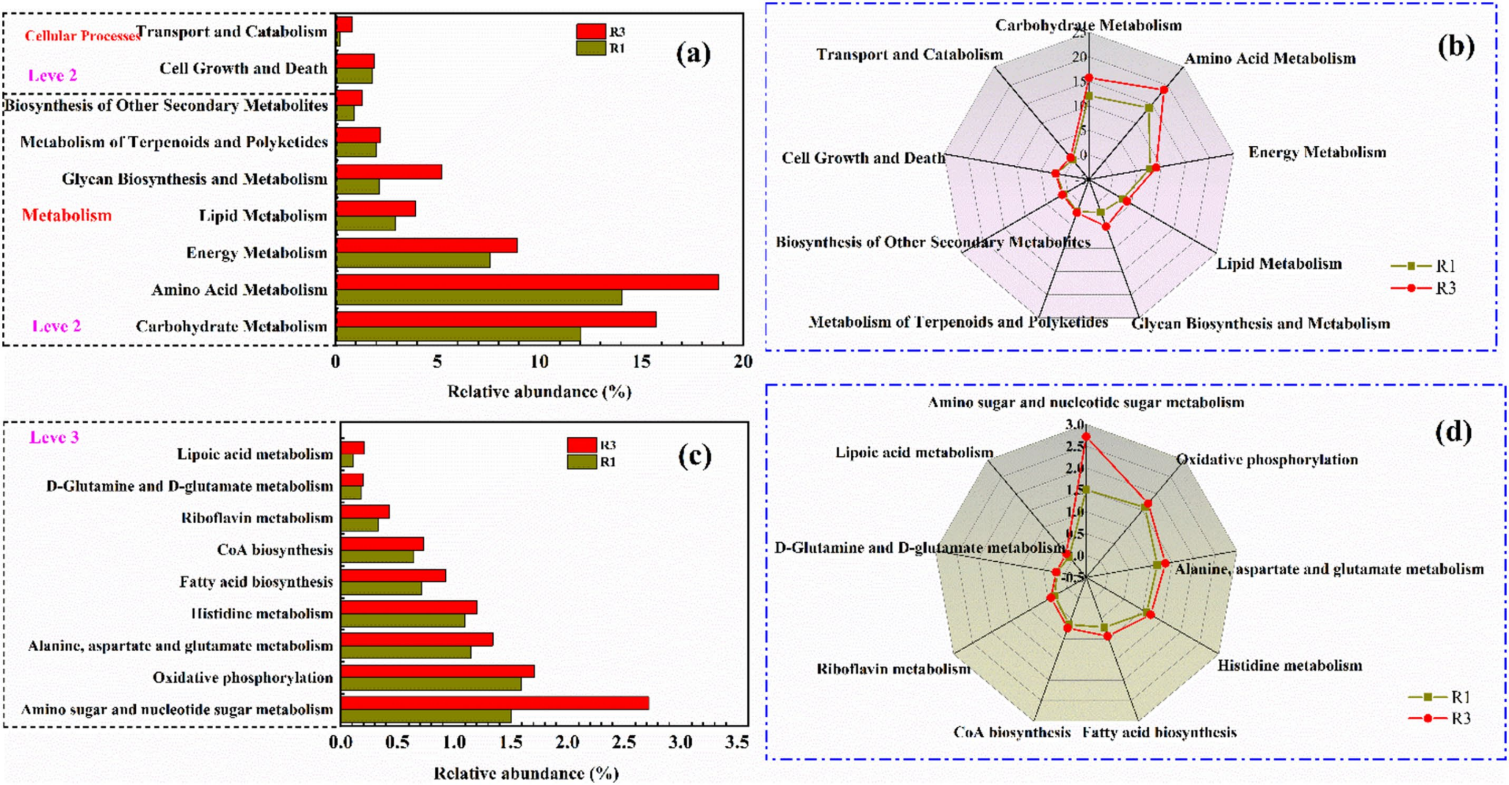
Functional predictions annotated of the KEGG database by Tax4fun, specifically level 2 KEGG functional predictions **(a,b)** and level 3 KEGG orthologue functional predictions **(c,d)**.

The catalog was juxtaposed with the KEGG databases to evaluate the significant functions of R1 and R3 in accordance with prior studies (Kanehisa and Sato, 2020; Kanehisa et al., 2021). More specifically, Figures 7a,b were annotated with functional predictions based on level 2 KEGG categories as determined by Tax4fun. The majority predicted functional genes involved in the chain elongation process under varying voltage conditions were categorized into metabolism and cellular processes. In the metabolic pathway, the abundance of gene associated with amino acid metabolism and carbohydrate metabolism was obviously enhanced under a voltage of 0.7 V. To be specific, the carbon metabolism of R3 increased by 30.42%, compared with R1. The carbohydrate metabolism of the chain elongation microorganisms exerted pronounced effects on caproate production and substrate consumption. Meanwhile, R3 increased the metabolism of amino acids by 33.66%, compared with R1. Amino acids are vital precursors for syntheses of protein with having enormous biological importance, such as growth, reproduction, and immunity. Besides, certain amino acids are referred to as functional amino acids, which encompass arginine, cysteine, glutamine, leucine, proline, tryptophan, and others (Liu et al., 2024; Ding et al., 2022; Jeon et al., 2022). In addition, energy metabolism refers to the flow and conversion of energy that occurs along with biological phenomena (Zhao et al., 2023; Chen et al., 2024). The R3 increases energy metabolism by 17.05%, compared with R1, suggesting that 0.7 V voltage strengthened the conversion of energy flow of chain elongation microorganisms.

The metabolic pathways of different treatments were analyzed at the level 3 (Figures 7c,d). Specifically, the results showed that the metabolism of amino acids (alanine, aspartate, and glutamate metabolism) and the biosynthesis of coenzyme A increased by 16.52 and 12.31% under 0.7 V, respectively. Among them, alanine, aspartate, and glutamate are often regarded as functional amino acids, which are the three essential amino acids for protein composition (Liu et al., 2024; Ding et al., 2022; Jeon et al., 2022). In addition, the biosynthesis of coenzyme A plays a crucial role in ethanol oxidation, FAB pathway, and RBO pathway. Based on the results, the optimal voltage of 0.7 V can enhance the metabolism of amino acids, carbon metabolism, energy metabolism, and coenzyme A synthesis, ultimately increasing caproate production.

Amino acid metabolism and carbon metabolism were accompanied by energy metabolism, which was one of the most fundamental characteristics of microorganisms. Through metabolic processes, microbial cells took up nutrients from the external environment, broken them down, and released the stored chemical energy, converting it into utilizable energy for tissues and cells. This energy was then used to sustain microbial life activities. The processes of energy release, transfer, storage, and utilization during material metabolism were collectively referred to as energy metabolism. In the carbon chain elongation process, carbon metabolism, amino acid metabolism, and energy metabolism were interdependent and indispensable. In summary, under the driving force of an applied voltage of 0.7 V, the oxidation of ethanol, the FAB pathway, and the RBO pathway were accelerated. The resulting H₂ and CO₂ were converted into acetate *via* the Wood-Ljungdahl (WL) pathway, which was then reused as a substrate for carbon chain elongation, preventing partial carbon loss. Processes such as ethanol oxidation, the FAB pathway, and the RBO pathway were accompanied by energy release, and the released energy was reused by microorganisms to sustain their life activities.

### 3.7 Metabolic pathways analysis of carbon chain elongation process

**Figure 8:**
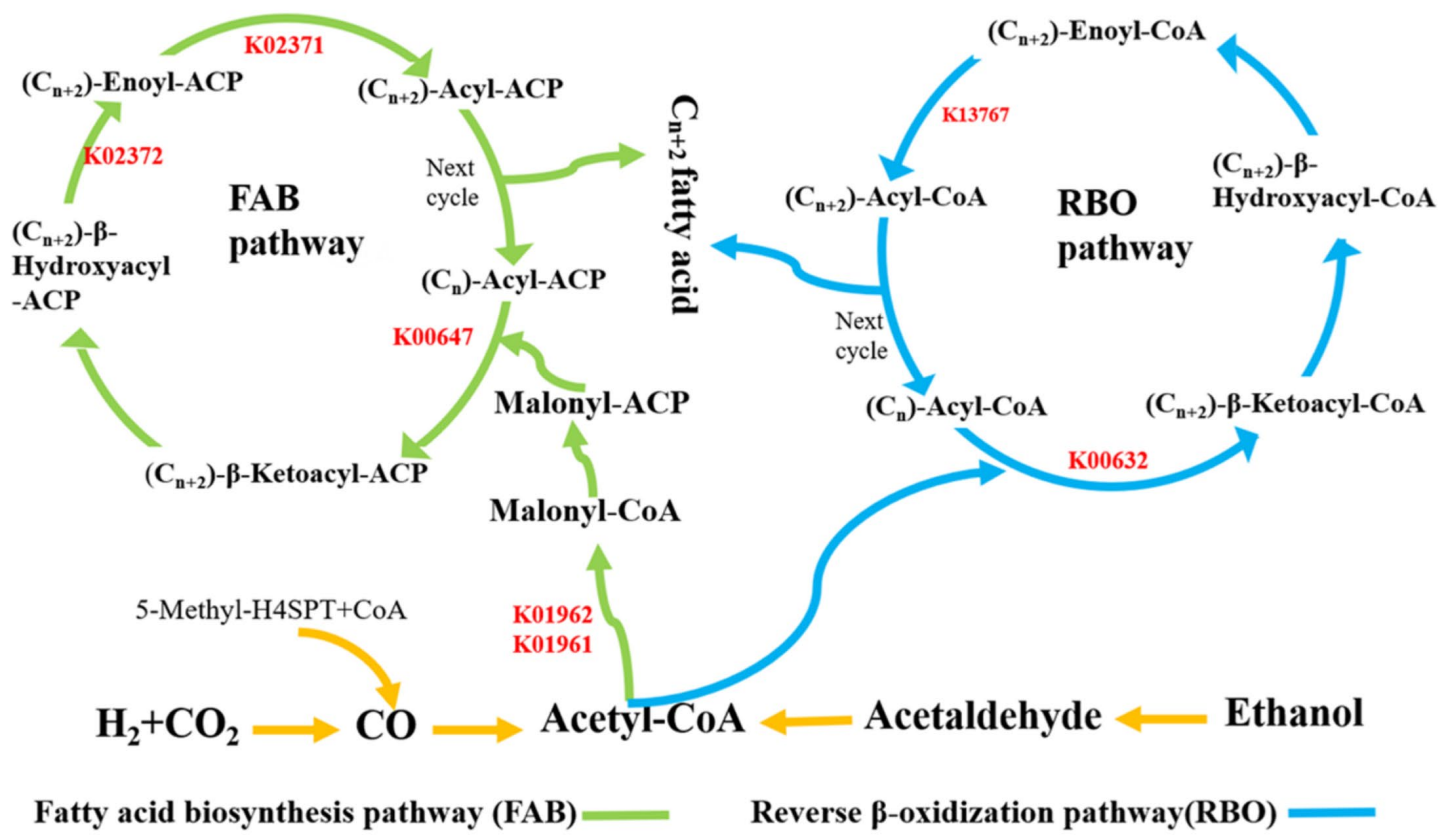
Tax4fun predicted chain elongation related metabolic pathway’s function.

**Figure 9:**
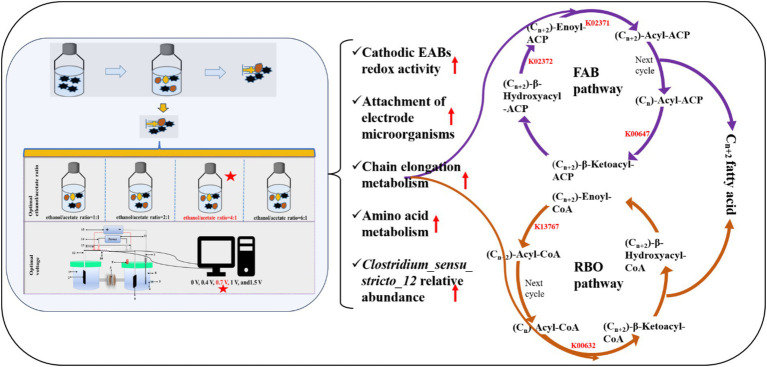
Carbon chain elongation reaction mechanism diagram.

Tax4fun was used to predict the function of metabolic pathways of the key microorganisms of carbon chain elongation process. In this study, the KOs associated with carbon chain elongation were screened and shown in Figures 8, 9. The results implied that the total of 7 KOs changes evidently from ethanol oxidation, RBO pathway, and FAB pathway under 0.7 V voltage regulation, including K01961, K01962, K00647, K02371, K02372, K00632, and K13767. The change of KOs existed in the following metabolic pathways.

Firstly, the RBO pathway was centered on acetyl-CoA with acetic acid as the electron acceptor. The cyclic reaction began with Acetyl-CoA and (Cn)-Acyl-CoA to form (Cn + 2)-*β*-Ketoacyl-CoA. In this process, K00632 changed evidently under 0.7 V. Later, (Cn + 2)-β-Ketoacyl-CoA was converted to (Cn + 2)-Acyl-CoA through a series of enzymatic reactions with the sharp change of K13767. Then, the carbon chain elongation process from Cn to Cn + 2 was achieved (Figures 8, 9).

Secondly, the FAB pathway was another carbon chain elongation pathway. The FAB pathway was centered on Acyl carrier protein (ACP), which was similar to acetyl-CoA in the RBO pathway. In the FAB pathway, Acetyl-CoA was converted into Malonyl-CoA by acetyl-CoA carboxylase, changing KOs, namely, K01961and K01962. Later, the Malonyl-ACP and (Cn)-Acyl-ACP formed the (Cn + 2)-β-Ketoacyl-ACP with the change of K0647. Subsequently, (Cn + 2)-β-Ketoacyl-ACP was further converted to (Cn + 2)-Acyl-ACP, changing KOs, namely, K02371 and K02372. Then, the Cn to Cn + 2 carbon chain elongation reaction was achieved (Figures 8, 9).

Based on the results, the group of 0.7 V significantly regulated 5 KOs in FAB pathway and 2 KOs in RBO pathway, indicating that the group with 0.7 V could promote both FAB pathway and RBO pathway.

## 4 Conclusion

In this work, the microbial enrichment of carbon chain elongation process was optimized with different carbon molar ratios of ethanol/acetate and voltages to the key parameters of carbon chain elongation process, aiming at having the optimal conditions of carbon chain elongation process. Finally, combined with microbial community analysis and functional prediction, the regulatory mechanism of synthesis of caproate in a carbon chain elongation process under optimal conditions was deciphered. The main conclusions are as follows:

(1) High carbon molar ratios of ethanol/acetate and high voltages are not conducive to the occurrence of the carbon chain elongation process. Under the initial pH of 7.00, the optimal ethanol/acetate carbon molar ratio and voltage were 4:1 and 0.7 V. In the optimal condition, caproate concentration increased by 83.09% in comparison to control group.
(2) Under the optimal conditions, the redox properties of cathode biofilm microorganisms and the colonization of microorganisms on the biofilm were enhanced. Meanwhile, it could also increase the relative abundance of microorganisms associated with carbon chain elongation: *Clostridium_sensu_stricto_12* (0.37–17.84%), *Clostridium_sensu_stricto_7* (0.09–2.49%), and *Christensenellaceae_R-7_group* (0.11−0.50%), which was conducive to acid production.
(3) In the optimal process, the metabolism of amino acids, carbon metabolism, energy metabolism, and coenzyme A biosynthesis were improved by 33.66, 30.42, 17.05 and 12.31% compared with the control group. Among them, the metabolism of amino acids mainly regulated the metabolism of alanine, aspartic acid, and glutamic acid. In addition, the optimal system seemingly presented well-regulated pathways of 2 KOs in the RBO and 5 KOs in the FAB. It was suggested that optimal system enhanced both FAB and RBO pathways.

## Data Availability

The original contributions presented in the study are included in the article/Supplementary material, further inquiries can be directed to the corresponding author.
