# Supplementary material for: Carbon chain elongation microorganism stimulates caproate production from ethanol and acetate under applied voltage regulation
Source: Front Microbiol. 2025 Jun 18;16:1597990. doi: 10.3389/fmicb.2025.1597990 (PMC12213838; doi:10.3389/fmicb.2025.1597990)
Supplement: Supplementary file 1 [file Supplementary_file_1.docx]

Supplementary Material

## Supplementary Figure S1

**Supplementary Figure S1.** The change of SEM images at the cathode biofilms with different treatments.

## Supplementary Figure S2

**Supplementary Figure S2.** The regional division map of EPS.
